# The Efficacy of Metformin as a Therapeutic Agent in the Treatment of Acne Vulgaris: A Systematic Review

**DOI:** 10.7759/cureus.56246

**Published:** 2024-03-15

**Authors:** Sherilyn Nguyen, Mai-Linh Nguyen, Will S Roberts, Michael Wu, Blake Smith, Tariq Rahaman, Hoang Nguyen

**Affiliations:** 1 Medical School, Nova Southeastern University, Fort Lauderdale, USA; 2 Library, Nova Southeastern University Dr. Kiran C. Patel College of Osteopathic Medicine, Clearwater, USA; 3 Basic Sciences, Nova Southeastern University Dr. Kiran C. Patel College of Osteopathic Medicine, Clearwater, USA

**Keywords:** acne, acne vulgaris, metmorfin, endocrine, controlled trials, polycystic ovary syndrome (pcos), dermatology

## Abstract

A large portion of the world's population is affected by acne vulgaris (AV), with many of these individuals being adolescents. The underlying mechanism of AV is hyperkeratinization and *Cutibacterium acnes* infection of the pilosebaceous follicle secondary to excessive stimulation of sebaceous glands by androgens. Metformin is a biguanide medication primarily used in efforts to lower patients' sugar levels in the management of type 2 diabetes. It has been proven to reduce levels of circulating androgens in patients with insulin resistance, indicating its potential for treating AV. A search strategy was developed and performed using the databases Ovid Medical Literature Analysis and Retrieval System Online (MEDLINE), Excerpta Medica database (EMBASE), Cumulative Index to Nursing and Allied Health Literature (CINAHL), Cochrane Controlled Register of Trials (CENTRAL), and Web of Science. The keywords “metformin” and “acne” were searched, along with related Medical Subject Headings (MeSH) and other subject headings. Studies that met the inclusion criteria were controlled trials, published after 2010, and in the English language. Participants with and without comorbidities such as polycystic ovary syndrome (PCOS) were considered. Two independent reviewers screened studies based on predefined criteria and extracted data from each study, which were quantitatively combined. A total of 15 studies were included in this systematic review. Across the 15 studies, there were 1,046 participants, with 13 studies looking exclusively at women with PCOS. Of the remaining two studies, one examined males with altered metabolic profiles, while the other included men and women with moderate AV. Notable risks of bias included studies that did not exclusively state the blindness of the study. Of the studies that were examined, 13 showed that metformin reduces AV, with seven studies showing statistical significance. Acne vulgaris is an inflammatory condition that has plagued patients for years due to the limited treatment options available. The hyperglycemic medication metformin, used in the management of type 2 diabetes, is being explored as a novel therapeutic that can possibly be repurposed for the treatment of AV. The use of metformin in AV is hypothesized to disrupt the proposed linkage between insulin resistance and AV proliferation. This proposed research could offer physicians a new option for the treatment of AV as well as render an alternative AV treatment for patients.

## Introduction and background

It is estimated that more than 9% of the world's population is affected by acne vulgaris (AV), with 85% of adolescents being affected [1]. The etiology of AV surrounds the response of the body’s sebaceous glands to circulating androgens, which, when increased, leads to both hyperkeratinization of the pilosebaceous unit and infection with *Cutibacterium acnes* [2]. Numerous modifiable and non-modifiable risk factors for the development of AV have been identified [2]. Debatably, the greatest risk factor for AV is comorbidity with polycystic ovary syndrome (PCOS) [3]. More than 75% of all patients with PCOS develop some level of AV, according to the National Institute of Health’s criteria for evaluating hyperandrogenism in the diagnosis of PCOS [3]. The criteria outline specific physical symptoms, such as increased AV growth and increased body hair growth. The significant spike seen in AV incidence is secondary to increased levels of circulating androgens in PCOS patients, triggering the aforementioned sequelae [3].

Metformin is a medication from the class of biguanides that has been utilized for the treatment of diabetes since the 1950s [4]. Metformin is a synthetic medication derived from galegine, which is a natural compound from the plant *Galego officinalis *[4]. The exact mechanism of action of metformin in the treatment of diabetes is unknown, but the logic behind its potential to treat AV is clear. Metformin reduces glucose levels, decreasing the risk of insulin resistance in patients with type 2 diabetes mellitus [5]. Insulin resistance is associated with increased secretion of luteinizing hormone (LH), which subsequently increases androgen secretion [5]. This interaction is compounded by patients with PCOS due to their significant predisposition to developing obesity, insulin resistance, and already elevated androgen levels [5].

It is estimated that more than 700 million people struggle with AV worldwide. While the mortality of AV is not very high, the burden it can place on individuals can be immense. Especially in severe cases, AV replaces the smooth facial skin with unpleasant erythematous cysts or comedones. If not treated appropriately, AV can lead to permanent scarring of the affected skin. With the prevalence of this condition being especially high in adolescents, the opportunity for AV to affect mental health cannot be understated. In 2013, more than one billion dollars was spent treating AV in the United States alone [6]. The extreme prevalence of this condition and the significant burden it places on patients both psychologically due to the stress they face in appearance in social settings and financially in an effort to find a treatment that works for them warrant the need for more therapeutics to combat AV.

## Review

Methods

**Figure 1:**
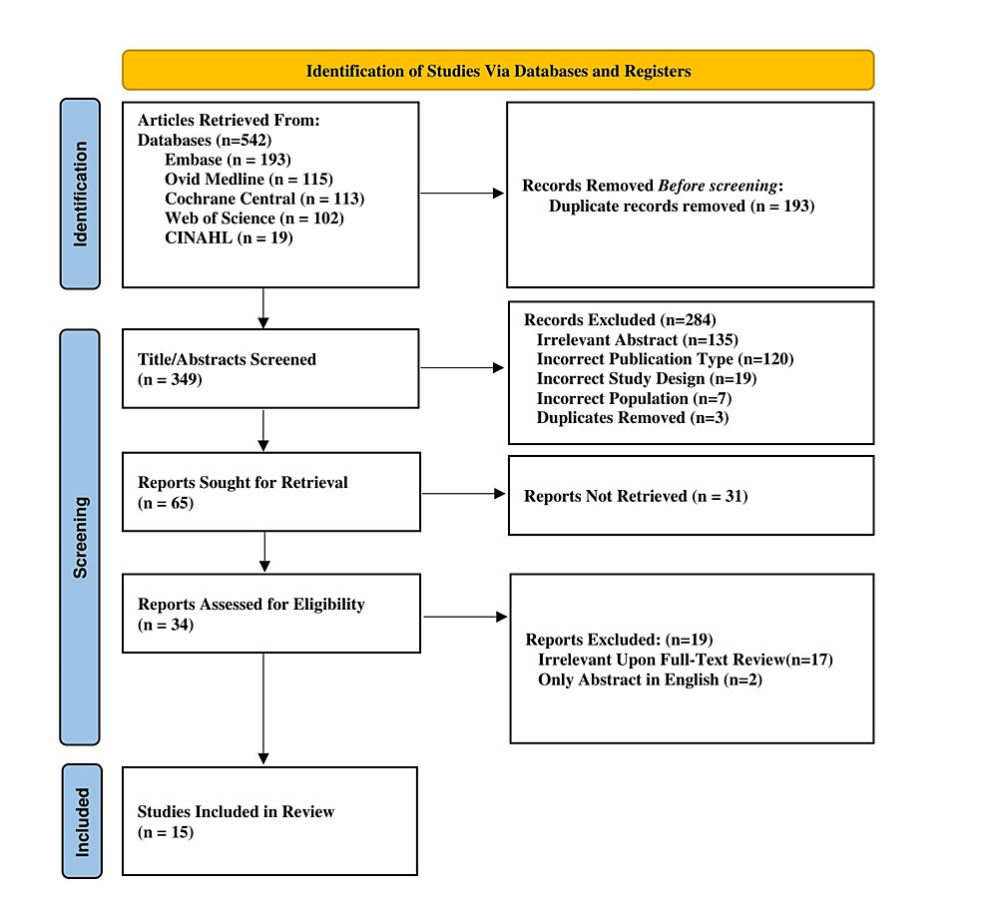
The PRISMA diagram encompassing the search strategy and screening methods of the studies for the systematic review EMBASE: Excerpta Medica database; MEDLINE: Medical Literature Analysis and Retrieval System Online; CENTRAL: Cochrane Controlled Register of Trials; CINAHL - Cumulative Index to Nursing and Allied Health Literature; PRISMA: Preferred Reporting Items for Systematic Reviews and Meta-Analyses

A search strategy was developed and performed using the following databases: Ovid Medical Literature Analysis and Retrieval System Online (MEDLINE), Excerpta Medica database (EMBASE), Cumulative Index to Nursing and Allied Health Literature (CINAHL), Cochrane Controlled Register of Trials (CENTRAL), and Web of Science. The keywords “metformin” and “acne” were searched, along with related Medical Subject Headings (MeSH) and other subject headings. The search strategies were developed by a medical librarian. Details of the search strategy can be found in Appendix 1, and the Preferred Reporting Items for Systematic Reviews and Meta-Analyses (PRISMA) diagram outlining the identification of the studies is shown in Figure 1.

Inclusion criteria

Only studies written in English and published between 2010 and July 2023 were considered for this systematic review. Only controlled trials involving human subjects were included (case reports, prospective studies, reviews, etc. were excluded). Participants with and without comorbidities (such as PCOS) were considered. Duplicate studies were excluded.

Study selection

Following the search, all identified citations were collated and uploaded into EndNote (Clarivate, London, UK). After a pilot test, titles and abstracts were screened by two independent reviewers for assessment against the inclusion criteria for the review. Potentially relevant studies were retrieved in full, and their citation details were imported into the Rayyan software (Rayyan Systems Inc., Cambridge, MA). The full text of selected citations was assessed in detail against the inclusion criteria by two independent reviewers. Any disagreements that arose between the reviewers at each stage of the selection process were resolved by an additional reviewer. The results of the search and the study inclusion process were reported in full in the final systematic review and presented in a PRISMA flow diagram (Figure 1) [7].

Assessment of methodology quality

All included studies, regardless of the results of their methodological quality, underwent data extraction and synthesis (where possible). Following critical appraisal, studies that did not meet a certain quality threshold were excluded. The critical appraisal score, generated by JBI (Faculty of Health and Medical Sciences, University of Adelaide, Adelaide, Australia), can be found in Table 1. The 13 assessment criteria are detailed in the description in Table 1. Following the assessment, JBI generated a score for each paper. Higher scores indicate higher-quality papers, while lower scores denote lower quality. Two reviewers then interpreted the scores and made final decisions to include or exclude papers. Any disagreements that arose between the reviewers at each stage of the selection process were resolved by an additional reviewer. No disagreements arose, and one study was excluded following the critical appraisal. At the end of the assessment, only one study was excluded.

**Table 1:** Critical appraisal of eligible randomized controlled trials This table depicts the quality assessment performed on all included studies to analyze the risk of bias. The assessment included 13 standardized criteria. Q: Question # Q1: Was true randomization used for the assignment of participants to treatment groups? Q2: Was allocation to treatment groups concealed? Q3: Were treatment groups similar at the baseline? Q4: Were participants blind to the treatment assignment? Q5: Were those delivering treatment blind to treatment assignment? Q6: Were outcome assessors blind to treatment assignment? Q7: Were treatment groups treated identically other than the intervention of interest? Q8: Was follow-up complete, and if not, were differences between groups in terms of their follow-up adequately described and analyzed? Q9: Were participants analyzed in the groups to which they were randomized? Q10: Were outcomes measured in the same way for treatment groups? Q11: Were outcomes measured in a reliable way? Q12: Was appropriate statistical analysis used? Q13: Was the trial design appropriate, and were any deviations from the standard randomized controlled trials design (individual randomization, parallel groups) accounted for in the conduct and analysis of the trial? Y: yes; N: no; U: undertermined

Citation	Q1	Q2	Q3	Q4	Q5	Q6	Q7	Q8	Q9	Q10	Q11	Q12	Q13
Altinok et al. [8]	Y	U	Y	U	U	U	Y	Y	Y	Y	Y	Y	Y
Elham et al. [9]	Y	Y	Y	U	U	U	Y	Y	Y	Y	Y	Y	Y
Wang et al. [10]	Y	N	Y	N	N	U	Y	Y	Y	Y	Y	Y	Y
DraveckÃ et al. [11]	Y	U	Y	U	U	U	Y	Y	Y	Y	Y	Y	Y
Bahadur et al. [12]	Y	U	Y	U	Y	N	Y	Y	Y	Y	Y	Y	Y
Tiwari et al. [13]	Y	Y	Y	Y	Y	Y	Y	Y	Y	Y	Y	Y	Y
Feng et al. [14]	Y	Y	Y	Y	Y	Y	Y	Y	Y	Y	Y	Y	Y
Fabbrocini et al. [15]	Y	Y	Y	U	U	U	Y	Y	Y	Y	Y	Y	Y
Tehrani et al. [16]	Y	Y	Y	Y	Y	Y	Y	Y	Y	Y	Y	Y	Y
Shahebrahimi et al. [17]	Y	Y	Y	U	U	U	Y	Y	Y	Y	Y	Y	Y
Seyam et al. [18]	Y	Y	Y	Y	Y	Y	Y	Y	Y	Y	Y	Y	Y
Sadati et al. [19]	Y	Y	Y	Y	Y	Y	Y	Y	Y	Y	Y	Y	Y
Rezai et al. [20]	Y	Y	Y	Y	Y	U	Y	Y	Y	Y	Y	Y	Y
Mhao et al. [21]	U	Y	Y	U	U	U	Y	Y	Y	Y	Y	Y	Y
Aqrawi et al. [22]	Y	N/A	Y	U	U	U	Y	U	U	U	U	U	U
Total %	93.3	66.7	100	40	46.7	33.3	100	93.3	93.3	93.3	93.3	93.3	93.3

Data extraction

Data were extracted from studies included in the review by one independent reviewer using the standardized JBI data extraction tool. The extracted data included specific details about the participants, study methods, interventions, and outcomes of significance to the review objective. The data extraction table generated by JBI can be found in Table 2 [23]. The extracted data reports multivariate p-values for the association between metformin use and AV severity. The specific population and experimental groups were also reported.

**Table 2:** Summarized results of the included studies OCP: oral contraceptive pill; Not all participants had polycystic ovary syndrome (PCOS); AMP: Altered metabolic profile which includes impaired fasting glucose, raised levels of total and low-density lipoprotein cholesterol, waist circumference, and BMI at the upper limit; Symptomatic anti-acne treatment included the use of bland detergent, azelaic acid, and nicotinamide.

Author, PY	Comorbidities	Population size	Intervention	Intervention duration	P-Value
Altinok et al. [8]	PCOS	90	A: metformin; B: metformin daily and OCPA; C: OCP	12 months	<0.05
Elham et al. [9]	PCOSB	70	A: metformin; B: oral isotretinoin	6 months	<0.05
Wang et al. [10]	PCOS	68	A: drospirenone, metformin, and lifestyle modifications; B: cyproterone acetate, metformin, and lifestyle modifications	6 months	<0.001
DraveckÃ et al. [11]	PCOS	39	A: alfacalcidol; B: alfacalcidol and metformin; C: metformin	6 months	0.867
Bahadur et al. [12]	PCOS	73	A: metformin with 1,000 IU vitamin D3; B: metformin with 4,000 IU vitamin D3	3 months	0.218
Tiwari et al. [13]	PCOS	66	A: exercise (placebo); B: metformin and exercise	6 months	0.475
Feng et al. [14]	PCOS	82	A: Diane-35; B: Diane-35 and metformin	3 months	0.00
Fabbrocini et al. [15]	AMP^c^	20	A: metformin and symptomatic anti-acne treatment; B: symptomatic anti-acne treatment^D^	6 months	<0.03
Tehrani et al. [16]	PCOS	80	A: metformin; B: metformin, calcium, and vitamin D; C: calcium and vitamin D; D: placebo	4 months	>0.05
Shahebrahimi et al. [17]	PCOS	56	A: metformin; B: pioglitazone	3 months	0.735
Seyam et al. [18]	PCOS	200	A: simvastatin and metformin; B: simvastatin; C: metformin	12 months	0.001
Sadati et al. [19]	None	40	A: doxycycline and benzoyl peroxide; B: metformin and benzoyl peroxide	2 months	<0.001
Rezai et al. [20]	PCOS	60	A: acarbose and clomiphene; B: metformin and clomiphene	3 months	0.11
Mhao et al. [21]	PCOS	26	A: metformin; B: ethinyl estradiol cyproterone acetate (EE-CA)	3 months	
Fruzzetti et al. [24]	PCOS	50	A: metformin; B: Myo-inositol plus folic acid	6 months	N/A

Results

Table 2 provides information on the author and publication year, comorbidities of the participants, population size, interventions used in the study, intervention duration, and p-values when available.

More details on each study can be found in Table 3. Studies included in the systematic review were controlled trials. A total of 15 studies were included in this systematic review. Thirteen studies showed AV improvement, as noted in Table 3. Of the studies that were included, 13 provided p-values. Seven studies found statistically significant improvements in the reduction of AV with metformin (Table 2). Across the 15 studies, there were 1,046 participants, with 13 studies looking exclusively at women with PCOS. The remaining two studies examined males with altered metabolic profiles, while the other included men and women with moderate AV. Seven studies noted that though metformin showed a reduction in AV, there was no statistical significance between metformin and the compared intervention. Two of the 15 included studies stated no improvement in AV with metformin treatment (Table 3). Four of the 13 studies that noted improvement in AV had p-values greater than 0.05, indicating no statistical significance even though a reduction in AV was seen. Eight studies had treatment durations of six months or more, while seven had treatment durations of four months or less. Of the seven statistically significant studies, five had treatment durations of six months or more, while two had treatment durations of four months or less, suggesting that the length of treatment could affect metformin’s ability to reduce AV. The age ranges that the 15 studies included greatly varied. As depicted in Table 3, three studies included participants in the adolescent age range, while two studies looked at early adulthood. Six studies included participants in both age ranges. Sixteen studies measured AV using objective measures such as the Global Acne Grading System (GAGS) and chi-square, as noted in Table 3. Nine studies used subjective measures such as clinical presence or questionnaires filled out by participants (Table 3).

**Table 3:** Characteristics of the included studies This table depicts the demographic information of the included studies, with population information and primary outcomes assessed. The number of participants with polycystic ovary syndrome (PCOS) was unspecified. OCP: oral contraceptive pill (OCP); GAGS: Global Acne Grading System (GAGS); AV: acne vulgaris; AMP: altered metabolic profile (AMP) which includes impaired fasting glucose, raised levels of total and low-density lipoprotein cholesterol, waist circumference, and BMI at the upper limit; IGA: Investigator Global Assessment; CADI: Cardiff Acne Disability Index (CADI); TLC: Total Acne Lesion Count

Study	Country	Setting Context	Population	Experimental Groups	Acne Vulgaris Measurement	Improvement vs No Improvement
Altinok et al. [8]	Denmark	12 months of treatment	90 women with PCOS; age range: 18-39 years old	A: metformin; B: metformin daily and OCP; C: OCP	PCOS-specific visual analogy scale questionnaire	Improvement
Elham et al. [9]	Iran	6 months of treatment	70 women with resistance acne and some with PCOS; age range: unspecified	A: metformin; B: oral Isotretinoin	GAGS and VisioFace® photography	Improvement
Wang et al. [10]	China	6 months of treatment	68 women with PCOS and metabolic disorders; age range: 16-22 years old	A: drospirenone, metformin, and lifestyle modifications; B: cyproterone acetate, metformin, and lifestyle modifications	GAGS	Improvement
DraveckÃ et al. [11]	Slovakia	6 months of treatment	39 women with PCOS; age range: unspecified	A: alfacalcidol; B: alfacalcidol and metformin; C: metformin	Clinical presence of AV	No improvement
Bahadur et al. [12]	India	3 months of treatment	73 women with PCOS; age range: 20-35 years old	A: metformin with 1,000 IU vitamin D3; B: metformin with 4,000 IU vitamin D3	GAGS	Improvement
Tiwari et al. [13]	India	6 months of treatment	66 women with PCOS; age range: unspecified	A: exercise (placebo); B: metformin and exercise	Presence of AV (6 months prior vs. 6 months during treatment)	No improvement
Feng et al. [14]	China	3 months of treatment	82 women with PCOS; age range: 26-32 years old	A: Diane-35; B: Diane-35 and metformin	GAGS	Improvement
Fabbrocini et al. [15]	Italy	6 months of treatment	20 young males with AMP and AV resistance to common therapy; age range: 17-24 years old	A: metformin and symptomatic anti-acne treatment' B: symptomatic anti-acne treatment	GAGS	Improvement
Tehrani et al. [16]	Iran	4 months of treatment	80 women with PCOS; age range: 20-40 years old	A: metformin; B: metformin, calcium, and vitamin D; C: calcium and vitamin D; D: placebo	Percent frequency of early forehead, cheek, and nose area involvement	Improvement
Shahebrahimi et al. [17]	Iran	3 months of treatment	56 women with PCOS; age range: 20-49 years old	A: metformin; B: pioglitazone	Percentage of PCOS patients experiencing AV	Improvement
Seyam et al. [18]	Egypt	12 months of treatment	200 women with PCOS; age range: unspecified	A: simvastatin and metformin; B: simvastatin; C: metformin	Unspecified “score”	Improvement
Sadati et al. [19]	Iran	2 months of treatment	40 men and women with moderate AV; age range: 15-40 years old	A: doxycycline and benzoyl peroxide; B: metformin and benzoyl peroxide	GAGS, IGA, CADI, TLC, and inflammatory and non-inflammatory lesion count	Improvement
Rezai et al. [20]	Iran	3 months of treatment	60 infertile women with PCOS; age range: 20-40 years old	A: acarbose and clomiphene; B: metformin and clomiphene	Percent frequency of AV	Improvement
Mhao et al. [21]	Iran	3 months of treatment	26 females with PCOS; age range: 14-40 years old	A: metformin; B: ethinyl estradiol cyproterone acetate (EE-CA)	AV improvement frequency	Improvement
Fruzzetti et al. [24]	Italy	6 months of treatment	50 women with PCOS; age range: 18-28 years old	A: metformin; B: myo-inositol plus folic acid	Subjective reporting by participants	Improvement

Seven studies examined metformin and other common AV treatments such as oral contraceptive pills (OCP), doxycycline, anti-androgens, and lifestyle modifications. Two studies looked at metformin as an adjunctive therapy; two studies compared metformin to other insulin sensitizers; one compared metformin to a statin; and three compared metformin to supplements, mainly vitamin D. These studies showed that metformin does reduce AV, and many of the studies found no significant difference between the interventions, suggesting that metformin is comparable to common AV treatments. Notable risks of bias include studies that do not exclusively state the blindness of the study. A critical appraisal of the 15 studies in the review can be seen in Table 1.

Discussion

The goal of this systematic review is to investigate the efficacy of metformin as a therapeutic agent in the treatment of acne vulgaris (AV). The findings of our research suggest a possible role for metformin in managing AV symptoms, which aligns with emerging literature examining the impact of this widely used antidiabetic medication. In total, 13 out of the 15 studies showed a reduction in AV, with seven of those studies indicating a statistically significant reduction in the severity of AV lesions among the metformin-treated group compared to the control group (Table 2).

Our findings propose a link between insulin resistance and the pathogenesis of AV. Metformin, by improving insulin sensitivity, may alter the underlying mechanisms that contribute to the exacerbation of AV. There are several possible pathways in which metformin is thought to be involved in the pathogenesis of AV. One proposed mechanism is through the reduction of insulin growth factor-1 (IGF-1) levels, which is a human growth factor found in sebocytes and sebaceous ducts that have been found to exhibit a strong positive correlation with facial sebum production and the occurrence of AV [25]. Elevated insulin levels are associated with higher IGF-1 levels [26]. Prior research has proposed a potential link between IGF-1 and the presence of AV by increasing the expression of pro-inflammatory biomarkers and activating inflammatory cascades within sebaceous glands [27]. In addition, IGF-1 directly affects the regulation of androgens in the skin through the activation of both the 5α-reductase and the androgen receptor [27]. Metformin acts on this pathway by activating altered metabolic profile (AMP)-activated protein kinase (AMPK), an enzyme involved in cellular homeostasis and energy expenditure [28]. The suppression of IGF-1 signaling has been linked to AMPK activation. The activation of AMPK by metformin is thought to set off a series of events that eventually decrease IGF-1-related pathways [25]. A decrease in IGF-1 can cause a reduction in androgenic hormones, and, thus, decrease the further progression of AV [25]. It is important to note that the relationship between metformin and AV is not yet fully understood. These mechanisms are current theories supported by experimental data; nonetheless, more research is required to give a more thorough understanding of how metformin works in the setting of AV. Furthermore, more complex variables, such as a patient’s individual response and specific AV subtype, may influence the link between metformin and AV treatment.

Six articles used the GAGS for the measurement of AV, as noted in Table 3. The Global Acne Grading System is a quantitative measurement for AV. It considers the type of lesions, such as comedones and papules, and the location of the lesion. This method of AV measurement has been noted to be more accurate, especially for PCOS patients, due to its objective criteria [29]. Investigator Global Assessment (IGA) is a qualitative measurement of AV that grades from 0 to four based on descriptive facial AV criteria [30]. The Cardiff Acne Disability Index (CADI) is a questionnaire designed to measure the quality of life for teens and young adults with AV [31]. Total Acne Lesion Count (TLC) is the earliest known AV measurement that involves counting the type and number of lesions [32].

Two articles used questionaries as a measurement for AV severity. In one article, participants were asked to self-report their AV into three categories: “no change", "slight improvement, or "significant improvement” [24]. A study by Altinok et al. constructed a questionnaire with six topics regarding discomfort with PCOS, one of which was AV. Participants in the study rated their discomfort on a horizontal line measuring 0-100 millimeters, with 0 millimeters indicating no discomfort and 100 millimeters indicating severe discomfort [8]. One study used VisioFace® photography to aid in the severity measurement of AV. This machine helps capture full-face photos to allow for a comprehensive assessment of the skin, which was later assessed by an expert dermatologist who was blinded to the study [9]. Four articles used qualitative measurements of AV [16, 17, 20, 21]. Measurements included a clinical examination for the presence and/or frequency of AV.

While this review suggests a positive association between metformin and AV improvement, it is important to acknowledge the limitations of this study that can impact the interpretation of the findings. This systematic review had inclusion criteria that reviewed studies within the last 10 years, which only warranted 15 included articles. In addition, not all studies used an objective scoring system such as the GAGS and instead used subjective measures such as surveys from participants about the interpretation of their own AV. Further research is warranted to explore the ideal dosage and duration of metformin therapy for subtypes of AV management for more personalized therapeutic approaches. Additional studies using objective measurements of AV will allow a more accurate and universally applicable analysis of AV. This approach would help reach a firm endorsement of the use of metformin as a therapeutic agent.

## Conclusions

Acne vulgaris is an inflammatory condition that has plagued patients for years due to the limited treatment options available. The proposed research displays a linkage between insulin resistance and AV as one of the mechanisms of disease. Treatments targeting this linkage have been described in recent literature and could potentially offer new therapeutic modalities for AV. Metformin, the hyperglycemic medication used in the management of type 2 diabetes, is being explored as a novel therapeutic that could be repurposed for the treatment of AV. This proposed research displays a linkage between metformin usage and AV improvement, potentially offering physicians a new option for the treatment of AV as well as rendering an alternative AV treatment for patients who have not responded to classic treatments. The efficacy seen warrants further research and exploration into the usage of metformin to identify the optimal dosage and duration in hopes of improving treatment options for AV.

## Appendices

Appendix 1

Search Strategy

This appendix details the search strategy used for each database.

**Table 4:** Excerpta Medica database (EMBASE): 193 articles screened

1	'metformin'/de
2	metformin:ab,kw,ti
3	#1 OR #2
4	'acne'/exp
5	acne:ab,ti,kw
6	#4 OR #5
7	#3 AND #6
8	'crossover procedure':de OR 'double-blind procedure':de OR 'randomized controlled trial':de OR 'single-blind procedure':de OR (random* OR factorial* OR crossover* OR cross NEXT/1 over* OR placebo* OR doubl* NEAR/1 blind* OR singl* NEAR/1 blind* OR assign* OR allocat* OR volunteer*):de,ab,ti
9	#7 AND #8

**Table 5:** Cumulative Index to Nursing and Allied Health Literature (CINAHL): 19 articles screened

1	(SU metformin) OR (TI metformin) OR (AB metformin)
2	(SU "acne vulgaris") OR (TI acne) OR (AB acne)
3	S1 AND S2
4	TX allocat* random* OR (MH "Quantitative Studies") OR (MH "Placebos") OR TX placebo* OR TX random* allocat* OR (MH "Random Assignment") OR TX randomi* control* trial* OR TX ( (singl* n1 blind*) OR (singl* n1 mask*) ) OR TX ( (doubl* n1 blind*) OR (doubl* n1 mask*) ) OR TX ( (tripl* n1 blind*) OR (tripl* n1 mask*) ) OR TX ( (trebl* n1 blind*) OR (trebl* n1 mask*) ) OR TX clinic* n1 trial* OR PT Clinical trial OR (MH "Clinical Trials+")
5	S3 AND S4

**Table 6:** Medical Literature Analysis and Retrieval System Online (MEDLINE): 115 articles screened

1	exp Metformin/
2	Metformin.mp.
3	#1 OR #2
4	exp Acne Vulgaris/
5	Acne.mp.
6	#4 OR #5
7	#3 AND #6
8	((randomized controlled trial.pt. or controlled clinical trial.pt. or randomized.ab. or placebo.ab. or drug therapy.fs. or randomly.ab. or trial.ab. or groups.ab.) not (exp animals/ not humans.sh.))
9	#7 AND #8

**Table 7:** Web of Science Core Collection: 102 articles screened

1	TS= Metformin
2	TS= Acne
3	#1 AND #2
4	TS= clinical trial* OR TS=research design OR TS=comparative stud* OR TS=evaluation stud* OR TS=controlled trial* OR TS=follow-up stud* OR TS=prospective stud* OR TS=random* OR TS=placebo* OR TS=(single blind*) OR TS=(double blind*)
5	#3 AND #4

**Table 8:** Cochrane Controlled Register of Trials (CENTRAL): 113 articles screened

1	SU metformin OR TI metformin OR AB metformin
2	TI acne OR AB acne OR SU “acne vulgaris”
3	#1 AND #2
